# Plasma miRNAs associated with cognitive impairment and brain hypometabolism in individuals with mild cognitive impairment

**DOI:** 10.1186/s12883-026-04758-z

**Published:** 2026-02-23

**Authors:** Maryam Faeed, Helia Hosseini, Azin Abdollahi, Mahsa Aziz, Hadis Darabi, Ahmadreza Sohrabi-Ashlaghi, Shaghayegh Farhadi, Minou Najar Nobari, Hamidreza Badakhshan

**Affiliations:** 1 School of Biology, College of Science, Tehran, Iran; 2 School of Medicine, Tehran University of Medical Sciences, Tehran, Iran; 3 Faculty of Pharmacy, Tehran University of Medical Sciences, Tehran, Iran; 4 Clinical Research Development Center, Najafabad Branch, Islamic Azad University, Najafabad, Iran; 5 School of Medicine, Shahid Beheshti University of Medical Science, Tehran, Iran; 6 Department of Physiology, Faculty of Medicine, Tehran Medical Sciences, Islamic Azad University, Tehran, Iran; 7 Department of Clinical Psychology, Faculty of Medical Sciences, Sanandaj Branch, Islamic Azad University, Sanandaj, Iran; 8 UCLA School of Dentistry, Los Angeles, CA USA; 9 School of Medicine, Shahid Beheshti University of Medical Sciences, Tehran, Iran

**Keywords:** Alzheimer’s disease, MicroRNAs, FDG-PET, Biomarkers, Brain hypometabolism

## Abstract

Detecting practical biomarkers to facilitate early diagnosis of Alzheimer’s disease (AD) before the onset of overt symptoms is crucial for clinicians to develop preventive and therapeutic strategies. There is substantial experimental evidence indicating the dysregulation of several microRNA (miRNA) expression levels in AD. They are known to play a crucial role in modulating critical intracellular signaling pathways associated with the core pathophysiological mechanisms of AD. In this cross-sectional study, we investigated the association of blood-based miRNAs as non-invasive, cost-effective biomarkers for the early stages of AD, focusing on their impact on cognitive decline and metabolism. All data were extracted from the Alzheimer’s Disease Assessment Scale (ADNI) database. A total of 152 subjects were categorized into mild cognitive impairment (MCI) and control groups based on their clinical dementia rating (CDR) scores, and significant differences between these cohorts were explored. Our objective was to establish correlations between miRNA levels and cognitive impairment tests, such as the Alzheimer’s disease assessment scale (ADAS), as well as hypometabolism indices using fluorodeoxyglucose (FDG)-positron emission tomography (PET) neuroimaging. Our findings revealed associations between hsa-miR-19a-3p and hsa-miR-16-5p with brain hypometabolism, and between hsa-miR-125b-5p and cognitive performance. While these observations suggest that specific miRNAs may reflect early AD-related changes, they require replication in larger, independent, biomarker-confirmed cohorts to assess their potential clinical utility.

**Supplementary Information:**

The online version contains supplementary material available at 10.1186/s12883-026-04758-z.

## Introduction

Alzheimer’s disease (AD), the leading cause of dementia, is a slowly progressive neurodegenerative disease [1]. The precise underlying mechanism of AD is unknown. However, the accumulation of beta-amyloid plaques outside the neurons and neurofibrillary tangles inside the neurons is widely regarded as the central pathological mechanism underlying the deterioration of neuronal function, ultimately resulting in cognitive impairment, dementia, and neuropsychiatric symptoms including aggression/agitation, mood change and hallucination [2, 3]. Mild Cognitive Impairment (MCI) is considered an intermediate stage between normal aging and the earliest phases of Alzheimer’s disease (AD). Detecting reliable biomarkers for MCI is particularly important, as it allows for earlier identification of individuals at risk of progressing to AD, potentially improving early intervention and treatment strategies [4].

The blood-based assessment of microRNAs (miRNAs) among AD patients has recently attracted the attention of researchers due to its non-invasive nature which is superior to invasive techniques such as cerebrospinal fluid (CSF) testing [5, 6]. miRNAs with a length of 18 to 25 nucleotides are single-stranded non-coding RNAs that act as post-transcriptional regulators of gene expression [7]. They play a crucial role in gene silencing by binding to the 3’ untranslated region (3’ UTR) of their target messenger RNA (mRNA). Once attached to their corresponding target, they either cleave the mRNA or interfere with the translation process, ultimately exerting their regulatory control [8, 9]. miRNAs exhibit cell type specificity and can be detected in both intracellular and extracellular environments [10]. In the extracellular environment, they are referred to as “plasma-based circulatory miRNAs,” and they can be protected from degradation by being encapsulated within extracellular vesicles or by binding to Argonaute proteins [11].

Nearly 70% of the documented miRNAs exist in the human brain, compared to other tissues [12]. They play a vital role in various biological processes, such as synaptic plasticity and neuronal development [13]. Any imbalance in miRNA regulation can be implicated in multiple diseases’ development and progression. Similarly, both upregulation, and downregulation of specific miRNAs in the brain tissue, CSF, and plasma of individuals diagnosed with AD have been reported to have a modulatory role in AD through several pathophysiological pathways, including amyloid-beta and tau pathology, synaptic plasticity disruption, neuroinflammation, and mitochondrial decline [14]. For instance, miR-20 and miR-107 are thought to be involved in regulating critical genes implicated in AD, such as amyloid precursor protein and beta-site amyloid precursor protein cleaving enzyme1 (BACE1), respectively, which exhibit dysregulation in individuals affected by AD [15, 16]. miRNAs found in CSF are a more reliable source for studying neurodegenerative diseases; however, they are protected by the blood-brain barrier. In contrast, miRNAs in blood are more accessible and cost-effective [17]. A range of miRNAs were identified in both CSF and blood samples. Most were detected in both fluids, while some were exclusive to blood, and a small fraction appeared only in CSF. Recent studies have highlighted the potential of plasma miRNAs in AD [18]. For instance, Kruger et al., showed examining plasma microRNA levels in addition to neuropsychological assessments, increases the accuracy of MCI to AD conversion prediction [19]. In another study, Liu et al. found that several plasma miRNAs were significantly associated with core Alzheimer’s disease biological markers, including amyloid load, tau pathology, and neurodegeneration. These miRNAs mapped to disease-relevant pathways and improved biomarker classification accuracy, underscoring their potential as accessible blood-based indicators of AD pathology [20].

Furthermore, hypometabolism is also recognized as a significant risk factor for AD and contributes to the deterioration of brain function and cognitive abilities [21]. The brain’s energy supply is already impacted by the natural aging process through changes in blood-brain-barrier penetration and cerebral blood flow, resulting in reduced uptake of essential nutrients [22, 23]. This effect becomes even more exacerbated in individuals with AD, which is evident from Fluorodeoxyglucose-positron emission tomography (FDG-PET) scans conducted on individuals with AD, revealing reduced glucose uptake and specific patterns of lobar hypometabolism [24]. FDG-PET hypometabolism captures neuronal metabolic dysfunction that occurs earlier than structural changes detectable by MRI, signaling neurodegeneration before measurable brain atrophy appears [25]. In addition, it provides greater statistical power for detecting disease related change than standard cognitive scores, making it a sensitive outcome in clinical trial settings [26]. Despite extensive studies conducted on molecular and neuroimaging biomarkers of AD, there is a noticeable lack of information regarding the association between miRNAs and metabolic changes in FDG-PET scans of Alzheimer’s patients. Vergallo and colleagues have evaluated the association between baseline brain-enriched miRNAs and neuronal metabolism in cognitively normal people with a subjective complaint of memory impairment [27]. However, to our knowledge, no study thus far has addressed this association in patients with cognitive impairment.

The current study aims to bridge the existing gap and provide valuable insights about the potential of plasma-based miRNA as non-invasive biomarkers associated with cognitive impairment. We hypothesized that the concentration of miRNAs might be associated with the observed hypometabolism in MCI patients. Therefore, we used the Alzheimer’s Disease Neuroimaging Initiative (ADNI) database to conduct a cross-sectional study to evaluate the potential association of baseline concentrations of miRNAs with neuronal metabolism detected by FDG-PET and observed cognitive characteristics. The extracted miRNAs represent the total circulating pool, including extracellular vesicle-associated, protein-bound, and cell-free miRNAs in plasma. No CSF miRNAs were analyzed in the present study.

## Materials and methods

### Participants

Data used in this article were obtained from the Alzheimer’s Neuroimaging Initiative (ADNI) database, accessed through (http://adni.loni.usc.edu). Under the leadership of Michael W. Weiner, MD in 2003, ADNI was established as a public-private partnership. Its primary objective is to evaluate the combined use of various imaging modalities, biomarkers, clinical assessments, and neuropsychological evaluations in measuring the progression of mild cognitive impairment (MCI) and early Alzheimer’s disease (AD). Briefly, in the ADNI study, participants from the United States and Canada aged between 55 and 90 underwent various assessments and longitudinal follow-ups. Exclusion criteria encompassed factors such as high Hachinski Ischemic Score, recent medication changes within four weeks prior to the study and the time for follow-up, less than six years of education, high scores on the Geriatric Depression Scale, and specific medication usage. In ADNI database, patients are classified as cognitively normal (CN), MCI, and AD based on criteria such as mini-mental state examination (MMSE) scores, Clinical Dementia Rating (CDR) scores, or the logical memory subtest of the Wechsler Memory Scale (WMS) [28]. Further details about the selection process and comprehensive information about the cohort can be found in other sources [29].

For this study, we included CN subjects and subjects with MCI from the ADNI 2 cohort, for whom all demographic data (gender, age and education plasma-derived miRNA, and neuropsychological assessments, including Alzheimer’s Disease Assessment Scale (ADAS) and CDR were all available. Additionally, we collected data from individuals who completed the baseline (screening visit) fluorodeoxyglucose (FDG) positron emission tomography (PET) scans from BAIPETNMRCFDG dataset. More specifically, data for FDG-PET were extracted from “ADNI-BAI-PET-NMRC-FDG.csv”; data for miRNAs were extracted from “UPENN-CSF-Plasma-miRNA-Data-[ADNI2].csv”; and data for cognitive assessments were extracted from ADAS-ADNIGO23.csv, MOCA.csv and CDR.csv. Eventually, a total number of 152 participants were included in the study, consisting of 77 CN, and 75 MCI subjects.

### FDG-PET image processing

Fully processed PET images by Dr. Koeppe’s team at the University of Michigan are available on the ADNI Laboratory on Neuroimaging (LONI) website. Taking a voxel-based approach to the data in NIFTI format using the computer packages SPM5 and SPM12 (http://www.fil.ion.ucl.ac.uk/spm*)*, investigators at Banner Alzheimer’s Institute performed additional pre-processing on the PET scans. This involved deforming each baseline PET image into the coordinate space of the SPM brain template, resulting in spatial normalization to the SPM template. The images were then re-smoothed by a 3D Gaussian kernel with the full width at half maximum of 12 mm. All FDG‑PET analyses were performed using global metrics only. The hypometabolic convergence index (HCI) and the statistical region of interest (sROI), derived from a voxel-based image analysis algorithm, are both global indices used in FDG-PET scans to evaluate cerebral hypometabolism in relation to AD which are the indices used in this study. The HCI characterizes the overall hypometabolism of glucose in the brain by integrating voxel-wise information into a single measurement cross-sectionally. It quantifies the extent to which an individual’s FDG-PET image matches the hypometabolism pattern observed in AD patients. On the other hand, the sROI is a global index specifically designed for tracking changes in the cerebral metabolic rate for glucose (CMRgl) over time. It involves the identification of specific regions of interest in the brain that are affected by AD (sROI_AD_) or MCI (sROI_MCI_) and comparing the glucose uptake within these regions to unaffected regions. By analyzing the decline in glucose uptake within these predefined regions, the sROI enables longitudinal assessment of metabolic changes associated with AD progression [30, 31].

### miRNA biomarkers

Plasma miRNA measurements were obtained from the ADNI Biomarker Core. Under fasting conditions, plasma was collected from participants, and RNA was isolated from it, using the Urine miRNA Purification kit (Norgen, Thorold, ON), following the manufacturer’s protocol. Although originally developed for urine samples, this kit was selected as part of the standard operating procedures developed by the ADNI study team for efficient RNA extraction from low-volume plasma. This method was validated and applied consistently across fluids to ensure compatibility and comparability.

The miRNAs were then converted into complementary DNA (cDNA) in the reverse transcription-quantitative PCR (RT-qPCR) using the Taqman Advanced miRNA cDNA synthesis kit (A28007, Thermo Fisher). 5 µL of the RT mix underwent a universal miR-amplification step consisting of 14 cycles (miR-amp). The resulting miR-amp was diluted with 1:10 nuclease-free water. Afterwards, 110 µL of the diluted cDNA was mixed with 220 µL Taqman Fast Advanced master mix and 110 µL nuclease-free water to prepare for q-PCR. In the q-PCR process, the prepared q-PCR mix was then loaded onto a custom TaqMan Low-Density Array (TLDA) card, which contained triplicate probes for 64 miRNAs. ADNI pre-selected a panel of 64 for analysis. No additional selection or filtering was performed by the present study prior to quality-control procedures. Two participant samples were processed on each TLDA card, with 100 µL of the first and second sample loading into the first and last 4 ports of the 384-well card respectively.

miRNA data were analyzed and processed by Oregon Health and Science University. First, quality control (QC) measures were conducted on the expression data (quantification cycle (Cq) values) obtained from the RT-qPCR by the QuantStudio software and miRNAs with insufficient data were filtered out. Afterwards, Cq values were normalized by utilizing a positive control miRNA (cel-miR-39-3P spike-in) and a combination of endogenous normalization reference control miRNAs (such as has-miR-16-5P). Wells with acceptable QC but Cq > 34 were considered censored values and given special handling using censoring-aware methods in the analysis. For this matter, a statistical method called Bayesian interval-censored regression was employed. This method estimated and compared the means of different groups (such as AD vs. healthy control) accounting for factors like donor sex, and age. The Cq value of 40 (the final PCR cycle) was treated as the zero expression, and for Cq values above 34, the true Cq value was estimated to fall within the interval between the observed value and 40.

The ADNI plasma miRNA panel consists of 64 pre-selected miRNAs chosen based on three main criteria: first, for their detectability in plasma, second, prior associations with Alzheimer’s disease or related neuropathological processes, and third, coverage multiple AD-related biological pathways [18]. After removing miRNAs with undetermined Cq values or missing/NULL values across samples, 48 miRNAs were retained for statistical analysis. A detailed list of excluded miRNAs and reasons for removal is provided in Supplementary Table S1.

### Cognitive assessments

In this study, cognition was assessed using ADAS and CDR which are valid tests for evaluating the severity of cognitive and non-cognitive behavioral dysfunctions in patients with dementia. The ADAS-Cognitive Subscale (ADAS-Cog), is a commonly used cognitive assessment that provides raw scores ranging from 0 to 70 [32]. Higher scores on this scale indicate more significant cognitive impairment. Recognizing certain limitations of the 11-item version of ADAS-Cog, a 13-item version was recommended as an alternative. The extended version includes two additional items: delayed word recalls and a number cancellation or maze task, expanding the score range to 0–85. For our study, we employed both 11-item and 13-item versions of ADAS-Cog to comprehensively evaluate cognitive function. Additionally, clinical dementia rating (CDR), measures and classifies cognitive and functional abilities related to AD and similar forms of dementia. This assessment examines six key domains: memory, orientation, judgment and problem solving, community affairs, home and hobbies, and personal care. Domains are assessed for degree of impairment on a 5-point scale, including 0 = none, 0.5 = questionable, 1 = mild, 2 = moderate, and 3 = severe [33].

### Statistical analysis

The statistical analyses were performed using SPSS26 software. All participants and variables included in the analyses had complete data, with no missing or undetectable values. First, we performed a comparison between plasma microRNA concentration at baseline in control and MCI groups, using the Mann Whitney U test. Normality of data distributions was assessed using the Shapiro–Wilk test. As several variables were not normally distributed, non-parametric methods including Mann–Whitney U tests and Spearman correlations were employed where appropriate. Additionally, we investigated the potential correlations of different plasma-derived miRNAs with sROI/HCI, ADAS, CDR score, gender, age, and education. Pearson’s correlation was used for normally distributed variables, while Spearman’s rank correlation was applied for non-normally distributed variables. The type of correlation used for each analysis is indicated in the Results section. Finally, to identify potential confounding covariates, multilinear regression (MLR) models were performed, with HCI as dependent variables and microRNAs as predictors, with gender, age, and education entered as covariates. Then, using a similar approach, we tested the association between plasma baseline miRNAs concentration and ADAS at baseline. This time ADAS was set as a dependent variable. All statistical elaborations were set significant at p-values < 0.05.

## Results

### Participant demographic and clinical characteristics

**Fig. 1:**
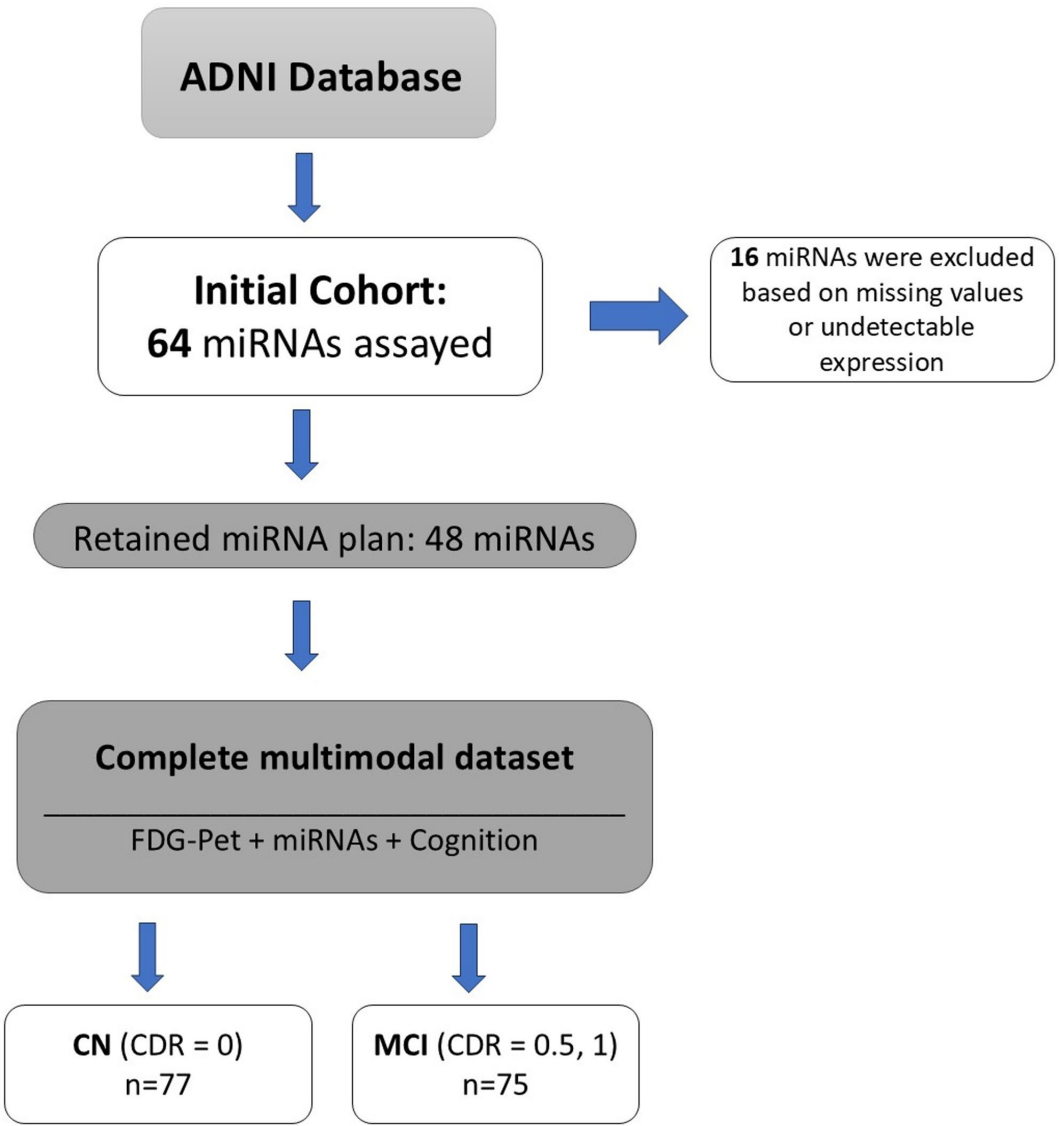
Participant flow diagram

A total of 152 patients, including cognitively normal (CN) and mild cognitive impairment (MCI) subjects, were included in this cohort study Fig. 1. The cohort consisted of 66 females and 68 males, with a mean age of 74.32 years. Demographic features, cognitive assessments, and FDG-PET measurements are reported in Table 1. The FDG-PET related indexes, HCI, SROI_AD_, and SROI_MCI_ differed between CN and MCI (*p* ≤ 0.001) and were significantly associated with ADAS cognitive tests. Mean expression levels for all 48 miRNAs in MCI and control groups are provided in Supplementary Table S2. However, no significant difference was observed between the two groups (*p* ≥ 0.00). Likewise, there were no significant differences in age and gender between the two groups. (*p* = 0.08 and *p* = 0.31, respectively). However, the years of education showed a significant difference between the two groups (*p* = 0.006).

**Table 1:** Demographic and clinical characteristics of the study

Number	Control (CDR = 0)	MCI (CDR = 0.5, 1)	*P* Value
	77	75	-
Gender (F/M)	37/40	29/46	0.3
Age	73.55 ± 6.87	75.12 ± 8.43	0.08
Education	16.42 ± 2.62	15.27 ± 2.46	**0.006**
ADAS-11	6.01 ± 3.43	21.21 ± 7.19	**≤ 0.001**
ADAS-13	9.47 ± 5.05	31.60 ± 8.30	**≤ 0.001**
HCI	8.61 ± 3.41	21.67 ± 7.33	**≤0.001**
SROI_MCI_	1.16 ± 0.06	1.02 ± 0.07	**≤ 0.001**
SROI_AD_	1.22 ± 0.05	1.09 ± 0.06	**≤ 0.001**

*Independent-Samples Mann-Whitney U Test on ranks test. Data are presented as mean ± standard deviation

*CN* cognitively normal, *MCI* mild cognitive impairment, *CDR* Clinical Dementia Rating, *ADAS*-13 Alzheimer’s disease Assessment Scale, *HCI* hypometabolic convergence index, *SROI* statistical region of interest. *p*-values < 0.05 are in bold

### microRNAs and hypometabolism

**Fig. 2:**
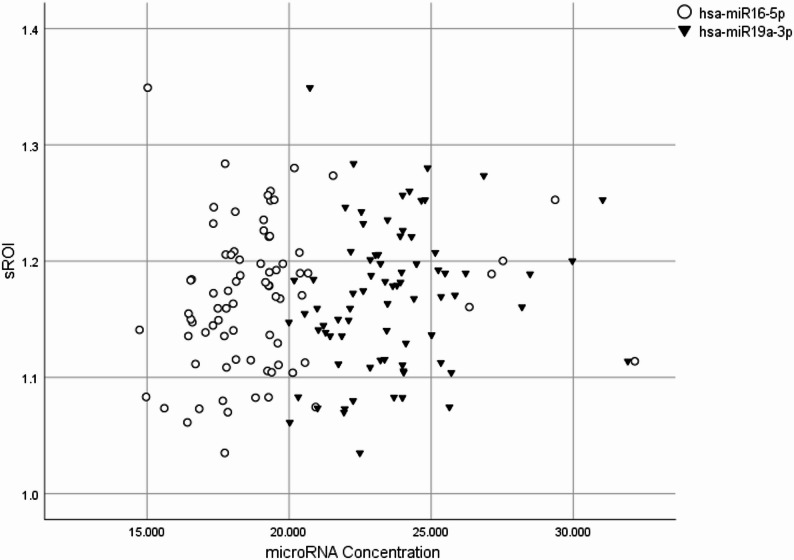
Scatterplot depicting the association between miRNAs concentration and FDG-PET indexes in the Control group. The positive correlation (*r* = 0.24) between SROI_MCI_ and hsa-miR16-5p and hsa-miR19a-3p are shown in circle and rectangle, respectively. sROI: statistical region of interest, MCI: mild cognitive impairment

The association of plasma baseline miRNA concentrations and brain hypometabolism was investigated. Among the CN group, hsa-mir16-5p and hsa-mir19a-3p were correlated with SROI_MCI_ (p-value < 0.05, *r* = 0.24; Fig. 2**)**. Importantly, these correlations reflect associations between the global FDG-PET indices (HCI and sROI), which integrate across multiple regions, rather than correlations with individual anatomical regions. No significant association between SROI_AD_ and the HCI index was found for other plasma miRNAs through Independent-Samples Mann-Whitney U Test. Further analysis through multiple linear regression failed to confirm any substantial connection between serum microRNA levels and hypometabolism in MCI groups.

### microRNAs and cognitive impairment

**Fig. 3:**
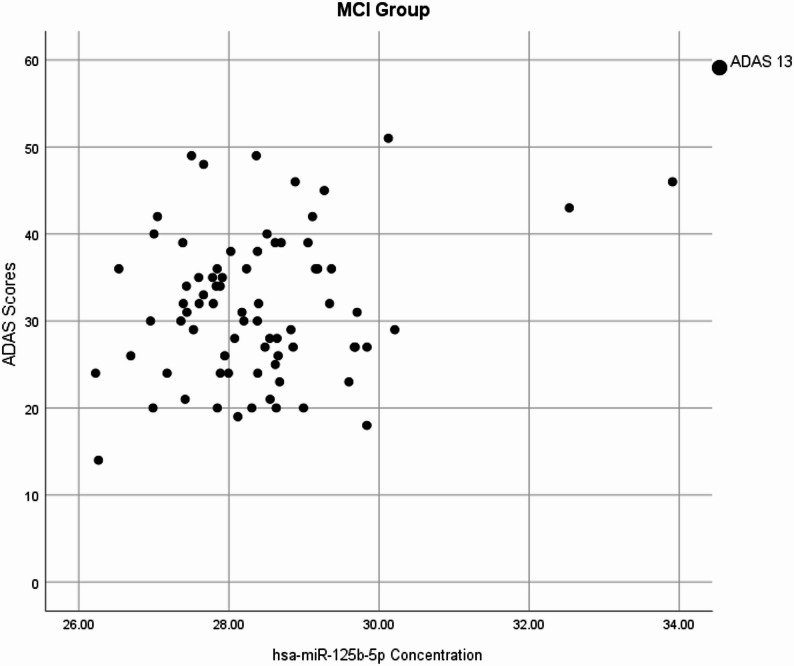
Positive Correlation of hsa-miR 125b-5p concentrations and ADAS 13 scores in MCI group. Higher hsa-miR-125b-5p levels were associated with better cognitive performance (*r* = 0.23)

**Fig. 4:**
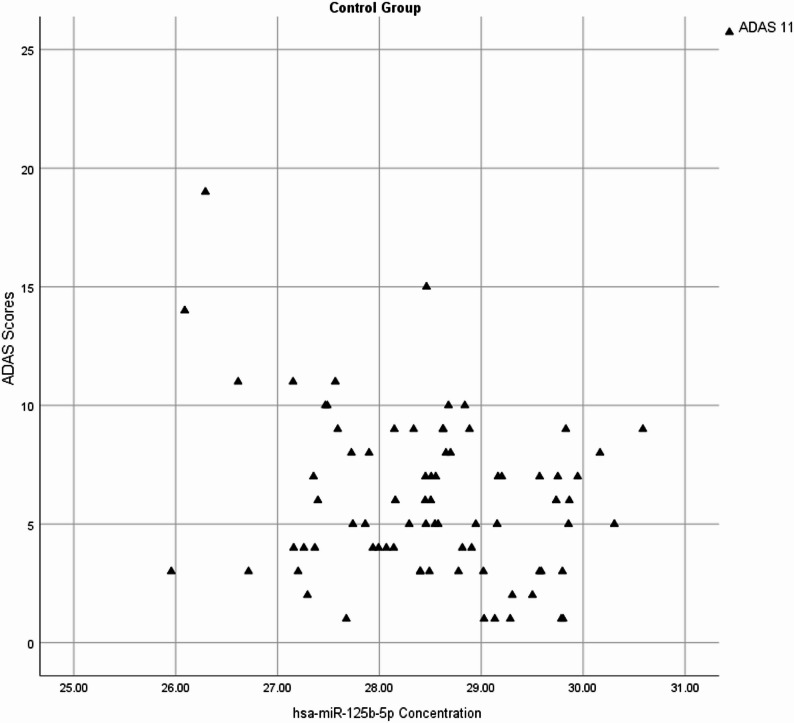
Negative Correlation of hsa-miR 125b-5p concentrations and ADAS 11 scores in CN group. Higher hsa-miR-125b-5p levels were associated with worse cognitive performance (*r*= -0.27)

We investigated the association between microRNAs and cognitive tests. In the MCI group, ADAS-13 positively correlates with hsa-miR125b-5p with (*p* = 0.04, *r* = 0.23; Fig. 3). However, in the control group, hsa-miR125b-5p negatively correlated with ADAS- 11 (p value = 0.018, *r*= -0.27; Fig. 4). A summary of the plasma miRNAs showing significant associations with cognitive decline or hypometabolism is provided in Table 2.

**Table 2:** Spearman’s correlations of neuropsychiatric assessments with FDG-PET hypometabolism indices

	CN				MCI			
	CDR		ADAS-13		CDR		ADAS-13	
	r_s_	*p*	r_s_	*p*	r_s_	*p*	r_s_	*p*
HCI	-	-	-0.162	0.160	0.266	0.021	0.553	<0.001
sROI_MCI_	-	-	-0.082	0.480	-0.363	0.001	-0.551	<0.001
sROI_AD_	-	-	-0.017	0.880	-0.411	<0.001	-0.566	<0.001

* *CN* cognitively normal, *MCI* mild cognitive impairment, *HCI* hypometabolic convergence index, sROI_MCI_ statistical region of interest affected by MCI, sROI_AD_ statistical region of interest affected by AD, *CDR* clinical dementia rating, *ADAS* alzheimer’s disease assessment scale, *P* – values < 0.05 are in bold

### Correlation of hypometabolism and cognition

CDR scores were correlated with hypometabolism indices including HCI, sROI_MCI_, and sROI_AD_ in MCI group (rs = 0.266, *p* = 0.021; rs = -0.363, *p* = 0.001; rs = -0.411, *p* < 0.001; respectively). We further studied the association between FDG-PET indexes and cognitive tests. In the MCI group, HCI positively correlated with ADAS-13 (p value = 0.00, *r* = 0.55), and ADAS-11 (p value = 0.00, *r* = 0.52). However, SROIMCI and SROIAD are negatively correlated with those tests (rs = -0.551, *p* < 0.001; rs = -0.566, *p* < 0.001; respectively). No such results were observed in the CN group Table 3.

**Table 3:** miRNA comparison

miRNA	Association with Hypometabolism	Association with Cognition	Group	Direction of Effect	Significant (p < 0.05)
hsa-miR-16-5p	Yes (SROI-MCI)	No	CN	Positive (r = 0.24)	Yes
hsa-miR-19a-3p	Yes (SROI-MCI)	No	CN	Positive (r = 0.24)	Yes
hsa-miR-125b-5p	No	ADAS-13 (MCI), ADAS-11 (CN)	MCI, CN	Positive (MCI), Negative (CN)	Yes

## Discussion

Alzheimer’s Disease (AD) is a prevalent neurodegenerative disease characterized by significant cognitive, physical, and social impairments. Therefore, the development of novel methodologies for its early detection is of paramount importance. Emerging evidence indicates the important role of microRNAs (miRNA) in the pathogenesis of AD [34]. Moreover, from a clinical perspective, miRNA profiling offers a minimally invasive and potentially cost-effective alternative to lumbar puncture and neuroimaging, facilitating scalable screening and longitudinal assessments [35]. To the best of our knowledge, the present study is first to comprehensively investigate the effect of plasma concentration of miRNAs on brain hypometabolism and cognitive impairment, which are hallmarks of AD. We evaluate the influence of an extensive array of miRNAs, including several being examined in the context of AD for the first time, thereby exploring their potential as precise and prognostic biomarkers for the disease.

Previous studies have demonstrated that only a limited set of miRNAs exhibited a significant relationship with age or gender, albeit after implementing statistical adjustments [27]. This observation aligns with the findings of our current study. Such a pattern may be ascribed to the recurrent discrepancies observed in miRNA profiles across separate studies targeting the same disease, as well as the inherent complexity and variable expression patterns of miRNAs within neurological disorders, in addition to technical challenges [36]. In our study, miR-30d-5p-Cq, miR-101-3p-Cq, miR-15a-5p-Cq, and miR-92b-3P-Cq were overexpressed in females. Nevertheless, to confirm the significance of the sex-dependent miRNA expressions and to mitigate gender bias in study analyses, it is imperative to employ larger cohorts and incorporate appropriate adjustments.

While it is established that miRNAs are dysregulated in various AD-affected brain regions, identifying the specific miRNAs linked to early-stage AD remains a subject of ongoing investigation. A multitude of dysregulated miRNAs have been detected in plasma samples of patients with mild cognitive impairment (MCI), providing a potential insight into disease progression [37]. Notably, due to shared functional characteristics among miRNA family members [38], four distinct miRNA families (miR-29, miR-30, miR-181, and miR-200) have exhibited significant associations with the progression from MCI to AD [14].

In the context of cognitive assessments, we found a significant positive association between miR-125b-5p and cognitive deficits as measured by the Alzheimer’s Disease Assessment Scale-Cognitive Subscale 13 (ADAS-13). This was consistent with another study showing its overexpression is associated with memory deficits. They also showed a miRNA signature that included miR-125b-5p predicted MCI-to-AD conversion [39]. A plausible explanation is that miR-125b, one of the most abundant miRNAs in the brain, is involved in the regulation of various processes in the central nervous system (CNS). These include tau phosphorylation, neuroinflammation, synaptic dysfunction, oxidative stress, neuronal survival and neural apoptosis, all of which are critical in the pathophysiology of neurodegenerative disorders. Furthermore, miR-125b’s influence on cognitive functions, particularly learning and memory, has been documented in various animal studies [40, 41]It is important to note that the literature presents conflicting evidence regarding the alterations in miR-125b-5p levels within dementia cohorts, with some studies reporting increases and others decreases [42, 43]. In addition, we showed negative correlation of hsa-miR-12-5b-5p with ADAS 11 in CN group, in contrast to MCI group. This pattern may suggest a stage-dependent role of miR-125b-5p in cognitive function: initially protective or compensatory, then becoming maladaptive as disease progresses, similar to biphasic patterns observed for other biomarkers such as microglial activation (initially protective, later neurotoxic) and neuronal activity. Moreover, ADAS-13 (used in MCI) includes additional items compared to ADAS-11 (used in CN), capturing different aspects of cognitive function. The opposite correlations could reflect that miR-125b-5p relates differently to memory versus executive function, or to different stages of cognitive decline captured by these distinct assessments. Validating this hypothesis requires longitudinal sampling to track within-individual trajectories, replication in biomarker-confirmed cohorts, and mechanistic studies in cellular/animal models directly testing whether miR-125b-5p manipulation affects the proposed pathways and cognitive outcomes.

It is noteworthy that hsa-miR19a-3p, hsa-miR5023p, hsa-miR2233p, and hsa-miR-1005p, generally (not specific to CN or MCI groups), yielded significant association with HCI upon regression model. However, in the control group, brain hypometabolism indexes have been correlated with two microRNAs only, miR-19a-3p and microRNA-16-5p [44]. A plausible explanation is that CN participants exhibit a more homogeneous metabolic profile, allowing subtle miRNA‑metabolism relationships to emerge, whereas the MCI group is biologically heterogeneous (varying degrees of amyloid, tau, and vascular burden) which can dilute associations [45]. Interpreting these associations is challenging, given that FDG-PET hypometabolism represents a downstream marker of neuronal dysfunction that is not AD-specific. Unlike amyloid and tau PET, which directly visualize AD pathology, glucose hypometabolism can result from various neurodegenerative and non-neurodegenerative processes [46]. Additionally, global indices (HCI, SROi) capture overall metabolic decline but cannot identify which neural circuits drive the effect, limiting mechanistic insight. Future work should incorporate regional or voxel‑wise or AD-specific analyses to refine biological interpretation.

Based on existing literature, we hypothesize that the observed correlation between miR-19a-3p and the statistical region of interest (sROI) could potentially be explained by this miRNA’s reported involvement in glucose metabolism pathways. It was found to be elevated during cerebral ischemic injury, and inhibiting its expression effectively reversed the suppression of glycolysis enzymes, glucose uptake, and neuronal apoptosis, influencing glucose metabolism [41]. miR-19a-3p modifies cell proliferation and axonal growth in embryonic cortical neurons [47]. This microRNA represses glucose uptake and promotes neural apoptosis and it has been shown to be related to the Wnt/β-catenin signaling pathway [48]. which is crucial for brain endothelial cell development and reportedly downregulated in AD. This pathway could theoretically affect Glucose transporter 1 (GLUT1), an integral membrane protein involved in glucose transportation [49]. However, direct investigation of the relationship between this miRNA and hypometabolism in AD is lacking, and our observed association requires experimental validation to determine whether these literature-reported mechanisms are relevant in the context of AD-related metabolic dysfunction.

The correlation we observed between miR-16-5p and sROI_MCI could potentially be related to various pathways implicated in AD pathogenesis, though this remains speculative without direct mechanistic evidence. An upregulation of this microRNA-16 family was reported in early AD stages versus late AD stages [50] suggesting its potential role in AD progression in early stages. Literature indicates this miRNA may interact with regulatory genes affecting autophagy and apoptosis of hippocampal neurons in AD [51]. Moreover, miR-16-5p has been implicated in the regulation of amyloid-β production and deposition through its influence on amyloidogenic processing pathways and can assist in preventing amyloid β-induced effects [52, 53]. Furthermore, has-miR-16-5p has been reported to modulate neurotrophic factors including brain-derived neurotrophic factor (BDNF) mRNA [54], a growth factor implicated in tau phosphorylation, Aβ accumulation, neuroinflammation, and neuronal apoptosis. By the progression of the disease, BDNF levels in the patient’s brain are reduced [55], though previous work found no significant concentration differences [54]. While our study identifies promising plasma miRNA candidates associated with AD-related brain hypometabolism and cognitive decline, these findings require validation in larger, independent cohorts, preferably with biomarker-confirmed AD, to strengthen their diagnostic and prognostic value. Improving technical reproducibility through standardized RNA extraction and quantification protocols will also be critical for broader application.

The lack of significant CN–MCI differences in plasma miRNA levels in our study, does not preclude biomarker relevance, as miRNAs may better reflect continuous metabolic processes than categorical diagnostic status. Diagnostic groups based on cognition can overlap substantially in underlying pathology, obscuring group-level differences. Plasma miRNAs may therefore be more informative when examined in relation to continuous metabolic measures rather than group comparisons. The observed group-specific associations may reflect false positives due to multiple testing, limited generalizability of the ADNI cohort, and the cross-sectional design. Heterogeneity within MCI and the absence of amyloid and tau PET data further limit interpretation. Accordingly, these findings should be considered exploratory and require validation in larger, longitudinal, and multimodal studies.

The rationale for focusing on plasma miRNAs stems from their upstream role in gene regulation, their presence in accessible biofluids, and their potential to reflect early molecular alterations not captured by conventional protein biomarkers. Importantly, plasma miRNAs may complement existing blood- and CSF-based biomarkers (e.g., amyloid, tau, neurofilament light) by capturing regulatory molecular mechanisms that proteins do not reflect, potentially enhancing early detection and disease monitoring accuracy [56]. Nonetheless, further work is needed to optimize assay sensitivity, establish clinical cutoffs, and evaluate cost-effectiveness in real-world settings.

## Conclusion

Considering the invasive nature of CSF-based biomarkers, there is increasing interest in accessible, blood-based alternatives. In this study, we examined the associations between plasma microRNA expression levels, FDG-PET hypometabolism, and cognitive performance. Our findings suggest that dysregulation of hsa-miR-19a-3p and hsa-miR-16-5p is associated with greater hypometabolism, potentially reflecting metabolic processes involved in neurodegeneration. Additionally, miR-125b-5p showed a positive relationship with cognitive performance in individuals with MCI. These findings support the potential role of plasma-derived microRNAs as early indicators of Alzheimer’s disease pathophysiology. However, further research in biomarker-confirmed AD cohorts is necessary to clarify the specific biological functions of these microRNAs and their utility in early detection.

### Limitations

This study has several limitations. First, it is cross-sectional in design, capturing data at a single time point rather than over time, and we did not examine whether MCI participants later converted to AD, therefore we cannot make claims about predictive biomarkers. Second, the number of participants with a confirmed Alzheimer’s disease diagnosis who completed all relevant assessments was too small to be included in the analysis. Additionally, the study sample consisted exclusively of American participants, which may limit the generalizability of the findings to more diverse ethnic populations. Group classifications (cognitively normal vs. MCI) were based on clinical dementia rating (CDR) scores rather than biomarker-confirmed AD pathology (e.g., amyloid or tau positivity), and the MCI group likely includes etiologically heterogeneous individuals, not all of whom may have underlying AD. Future studies utilizing biomarker-defined classifications based on the AT(N) framework may offer improved diagnostic specificity and yield more targeted insights into the role of plasma miRNAs in early AD. Another limitation is the absence of correction for multiple comparisons (e.g., Bonferroni or false discovery rate), which increases the risk of type I error; this reflects the exploratory nature of our study, and future confirmatory analyses should apply appropriate statistical corrections. We also did not assess the combined predictive power of miRNAs using machine learning models or cross-validation techniques (e.g., k-fold or leave-one-out), which could strengthen predictive robustness, this remains a priority for future work with larger, better-powered datasets. Lastly, our imaging analysis examined whole-brain metabolism rather than specific regions of interest; future studies with larger samples and extended follow-up may provide a more nuanced understanding of the dynamic role of these miRNAs in cognitive decline and metabolic changes.

## Supplementary Information

Supplementary Material 1.

## Data Availability

Data for FDG-PET were extracted from “ADNI-BAI-PET-NMRC-FDG.csv”; data for miRNAs were extracted from “UPENN-CSF-Plasma-miRNA-Data-[ADNI2].csv”; and data for cognitive assessments were extracted from ADAS-ADNIGO23.csv, MOCA.csv and CDR.csv. The analyzed datasets during the current study are available upon request with no restriction.
