# Supplementary material for: Plasma miRNAs associated with cognitive impairment and brain hypometabolism in individuals with mild cognitive impairment
Source: BMC Neurol. 2026 Feb 23;26:200. doi: 10.1186/s12883-026-04758-z (PMC13032452; doi:10.1186/s12883-026-04758-z)
Supplement: Supplementary file 1 — Supplementary Material 1. [file 12883_2026_4758_MOESM1_ESM.docx]

**Supplementary Table 1. Excluded miRNAs**

| miRNA | Reason for Exclusion |
| --- | --- |
| miR-323a-3p | Missing value |
| miR-484 | Missing value |
| miR-532-5p | Missing value |
| miR-660-5p | Missing value |
| miR-210-3p | Missing value |
| miR-146a-5p | Missing value |
| miR-204-5p | Missing value |
| miR-194-5p | Missing value |
| miR-19b-3p | Missing value |
| miR-192-5p | Missing value |
| miR-193a-5p | Missing value |
| miR-30b-5p | Missing value |
| miR-885-5p | Missing value |
| miR-499a-3p | Undetermined (except for 3 participants) |
| miR-647 | Undetermined |
| cel-miR-39 | Exogenous spike-in control, not analyzed |

**Supplementary Table 2. miRNAs Differences Across MCI and CN.** The differences are nonsignificant

| MicroRNA | CN | MCI |
| --- | --- | --- |
| hsa-let7b5p_Cq | 27.85 | 27.82 |
| hsa-mir223-3p_Cq | 19.81 | 19.21 |
| hsa-mir22-3p_Cq | 20.09 | 20.06 |
| hsa-mir23a-3p_Cq | 21.6 | 21.28 |
| hsa-mir26a-5p_Cq | 22.05 | 21.63 |
| hsa-mir26b-5p | 20.4 | 20.11 |
| hsa-mir27b-3p | 22.62 | 22.35 |
| hsa-mir29a-3p | 24.14 | 24.04 |
| hsa-mir29b-3p | 23.88 | 23.8 |
| hsa-mir29c-3p | 23 | 22.95 |
| hsa-mir30a-3p | 28.32 | 28.31 |
| hsa-mir30d-5p | 23.84 | 23.82 |
| hsa-mir338-3p | 27.17 | 26.93 |
| hsa-mir2110 | 27.12 | 27.28 |
| hsa-mir342-3p | 25.9 | 25.7 |
| hsa-mir374b-5p | 26.3 | 25.93 |
| hsa-mir376a-3p | 27.23 | 26.97 |
| hsa-mir378a-3p | 26.85 | 27 |
| hsa-mir423-5p | 22 | 22.23 |
| hsa-mir424-5p | 24.93 | 25.04 |
| hsa-mir425-5p | 24.36 | 24.23 |
| hsa-mir502-3p | 26.66 | 26.9 |
| hsa-mir92a-39 | 20.21 | 20.25 |
| hsa-mir214-3p | 28.83 | 28.85 |
| hsa-mir21-5p | 19.82 | 19.84 |
| hsa-mir181b-5p | 26.91 | 26.89 |
| has-let7g-5p | 24.95 | 24.79 |
| hsa-let7-i5p | 21.56 | 21.49 |
| hsa-mir100-5p | 29.78 | 29.93 |
| hsa-mir101-3p | 22.26 | 22.53 |
| hsa-mir125b-5p | 28.48 | 28.37 |
| hsa-mir142-3p | 22.43 | 22.01 |
| hsa-mir143-3p | 24.8 | 24.57 |
| hsa-mir145-5p | 24.09 | 23.94 |
| hsa-mir150-5p | 24.17 | 24.26 |
| hsa-mir153-3p | 29.98 | 29.77 |
| hsa-mir15a-5p | 20.28 | 20.34 |
| hsa-mir16-5p | 19.01 | 18.76 |
| hsa-mir181c-5p | 25.41 | 25.22 |
| hsa-mir200a-3p | 28.94 | 28.9 |
| hsa-mir19a-3p | 23.59 | 23.18 |
| hsa-mir199a-5p | 24.86 | 24.58 |
| hsa-mir195-5p | 26.39 | 26.14 |
| hsa-mir92b-3p | 20.19 | 20.29 |
| hsa-mir190a-5p | 30.08 | 29.99 |
| hsa-mir186-5p | 24.43 | 24.15 |
| hsa-mir185-5p | 23.04 | 22.96 |
| hsa-mir24-3p | 23.14 | 22.82 |
